# Glucose improves object-location binding in visual-spatial working memory

**DOI:** 10.1007/s00213-015-4125-5

**Published:** 2015-11-18

**Authors:** Brian Stollery, Leonie Christian

**Affiliations:** School of Experimental Psychology, University of Bristol, 12a Priory Road, Bristol, BS8 1TU UK

**Keywords:** Glucose, Episodic memory, Spatial memory, Working memory, Object-location memory, Binding, Complexity, Hippocampus, Human

## Abstract

**Rationale:**

There is evidence that glucose temporarily enhances cognition and that processes dependent on the hippocampus may be particularly sensitive. As the hippocampus plays a key role in binding processes, we examined the influence of glucose on memory for object-location bindings.

**Objective:**

This study aims to study how glucose modifies performance on an object-location memory task, a task that draws heavily on hippocampal function.

**Methods:**

Thirty-one participants received 30 g glucose or placebo in a single 1-h session. After seeing between 3 and 10 objects (words or shapes) at different locations in a 9 × 9 matrix, participants attempted to immediately reproduce the display on a blank 9 × 9 matrix. Blood glucose was measured before drink ingestion, mid-way through the session, and at the end of the session.

**Results:**

Glucose significantly improves object-location binding (*d* = 1.08) and location memory (*d* = 0.83), but not object memory (*d* = 0.51). Increasing working memory load impairs object memory and object-location binding, and word-location binding is more successful than shape-location binding, but the glucose improvement is robust across all difficulty manipulations. Within the glucose group, higher levels of circulating glucose are correlated with better binding memory and remembering the locations of successfully recalled objects.

**Conclusions:**

The glucose improvements identified are consistent with a facilitative impact on hippocampal function. The findings are discussed in the context of the relationship between cognitive processes, hippocampal function, and the implications for glucose’s mode of action.

Glucose is the major energy substrate supporting neuronal functioning (Messier 2004), and it is known that the central nervous system, particularly the hippocampus, is especially vulnerable to interruptions in its supply (Dennis et al. 2011; Tomlinson and Gardiner 2008). Recent comprehensive reviews have concluded that drinking glucose, following an overnight fast, can produce a temporary enhancement of cognition that is often, although not exclusively, seen on episodic memory tasks or cognitive tasks that pose a high level of demand (see Messier 2004; Riby 2004; Smith et al. 2011). While the favoured status of episodic memory can be interpreted in terms of a hippocampal specific action (e.g. McNay and Gold 2001; Riby and Riby 2006; Winocur 1995), only a few human studies have undertaken a focussed evaluation of hippocampal-based cognitive tasks to explore this (e.g. Stollery and Christian 2015).

Given the special status afforded to the hippocampal system for understanding the influence of glucose on cognition, the present study explores glucose’s influence on a task widely acknowledged to draw on hippocampal functioning: the object-location memory task. In this task, participants maintain in working memory a spatial array of objects (e.g. letters, pictures, or colours) for retrieval. Memory for the objects, the locations of the objects, and the binding of object-location pairs are key performance parameters. The latter memory index is particularly pertinent because an important role of the hippocampus during the creation of episodic memories are the binding processes that integrate diverse stimulus attributes (e.g. colour, shape, size, location) into unitary representations (e.g. Mitchell et al. 2000). Indeed, forming and maintaining bound representations is a common activity of the cognitive system (Zimmer et al. 2006) and, as our visual environment contains many objects at different locations, object-location memory is the key feature of working memory enabling us to keep track of the whereabouts of objects in our world (e.g. where is my phone?) and is regarded as the basic binding problem (Treisman 1996).

As the name implies, object-location memory entails remembering what went where and processing visually presented material is organized into two broad functional pathways. The ventral, or “what”, pathway is important for visual object recognition and the dorsal, or “where”, pathway for the localisation of objects in space (Mishkin and Ungerleider 1982; Ungerleider and Haxby 1994) and one key question concerns the integration of the what and where information streams. Given these two pathway connections to the limbic system and frontal lobes, Mishkin et al. (1983) speculated that one plausible site for this integration is the hippocampal formation. Since that time a range of studies offer broad support for the view that distinct, but interacting (McIntosh and Schenk 2009), neural pathways are involved in remembering object and position information (e.g. Moscovitch et al. 1995; O'Keefe and Nadel 1978) and that hippocampal structures play a critical role in the binding of objects to locations (Bachevalier and Nemanic 2008; Crane and Milner 2005; Finke et al. 2008; Gilbert and Kesner 2004; Hannula and Ranganath 2008; Mumby et al. 2002; Nunn et al. 1999; Olson et al. 2006; Pertzov et al. 2013; Piekema et al. 2006; Postma et al. 2008; Watson et al. 2013). These conclusions are not surprising given the fundamental role of hippocampal structures to relational memory (e.g. Cohen et al. 1999; Olsen et al. 2012, 2013); although, in common with other forms of episodic memory, object-location memory recruits additional neural systems (e.g. prefrontal cortex) to support efficient functioning (see Aggleton 2012; Barker et al. 2007; Barker and Warburton 2015; Lee and Solivan 2008).

Only a few studies have examined the influence of glucose on tasks relevant to the object-location memory. Benton and Owens (1993)showed 16 pictures (e.g. cat, doll) in a 4 × 4 grid for 30 s and after 1 min of rehearsal-preventing activity, participants relocated the 16 pictures. They found that a 50-g glucose dose did not influence the speed or accuracy of replacing the pictures. Using the same technique, two related studies report better performance with higher blood glucose levels, with the pictures being located faster and more accurately (Benton and Parker 1998; Benton and Sargent 1992). A later study by Benton and Stevens (2008) examined 25 g glucose with children aged 9–10 years. After showing 20 pictures of common objects (e.g. ball, mouse) on a card, the children free-recalled the names of the objects shown. After presenting the same card twice more, with name recall requested after each presentation, the children attempted to relocate the 20 pictures in an empty 5 × 4 grid. Those receiving glucose recalled more objects (averaged over the three trials) but did not differ in their placement of the objects. Mohanty and Flint Jr. (2001) presented 16 pictures (all either negative or neutral valence) in a 4 × 4 grid for 20 s and, following 1 min of rehearsal prevention, participants attempted to place the pictures back in their correct location. This procedure was repeated twice more, using the same picture arrangement, and recall scores averaged. Those given 50 g glucose took longer to replace the pictures and made more placement errors with the emotional material, but glucose did not influence the performance for the neutral material. In all the above studies, a separate assessment of object memory and location memory is unavailable because all locations contained objects and all the objects were available to the participant during the replacement phase. Thus, the evaluations only consider the correct placement of objects.

Scholey and Kennedy (2004) and Jones et al. (2012) examined location memory by showing participants a house with nine windows, four windows of which were “lit”, and tested recognition memory for lit and unlit windows. Unfortunately, by amalgamating different aspects of performance into composite scores (e.g. quality of memory, speed of memory), this study did not evaluate explicitly the effect of glucose on location memory. However, based on the composite scores that incorporate performance on this spatial memory task, glucose either did not influence performance (Scholey and Kennedy 2004) or produce impairment (Jones et al. 2012). Stollery and Christian (2013) also only examined location memory by presenting identical objects (circles) at random locations in a 9 × 9 grid and assessing recognition memory for exact locations. Although location memory declines substantially as the number of objects to remember increases, neither speed or accuracy varied with the 50 g glucose dose given. However, they observed a trend for a slower decline in recognition accuracy with increasing memory load for those given glucose with a drink congruent message.

Finally, three glucose studies have used a computerized version of the Corsi blocks task. Although this task clearly requires location memory, the requirement to reproduce the temporal sequence of locations places additional demands on visual-spatial working memory (Zimmer et al. 2003). As a spatial variant of the verbal digit-span task, typically, evaluations of temporal-spatial memory assess both forward and backward spans (Berch et al. 1998; Brunetti et al. 2014), but glucose studies have only focussed on forward span. Sünram-Lea et al. (2011) found that 25 g glucose improved performance, but lower (15 g) and higher (50 and 60 g) doses did not. Owen et al. (2012) failed to observe any influence of either a 25- or 60-g glucose dose, following either an overnight fast or a 2-h fast, although a later study found improved spans for both a 25- and 60-g glucose dose following an overnight fast (Owen et al. 2013).

Taken together, there is rather heterogeneous evidence for an impact of glucose on the cognitive processes relevant to object-location memory. Moreover, when all locations are used, and participants given the entire set of objects to relocate, the study is unable to evaluate the separate roles of object memory and location memory in successful object-location binding. Additionally, as all locations contain objects, an error in the placement of one object will always lead to an error in the placement of the object originally at that location. More importantly, the sensitivity of the relevant studies to detect glucose related effects is likely to be low for two reasons. First, most studies of object-location memory only use a single presentation-recall trial and the resulting estimates of memory are likely to be less stable compared to those achieved from multiple trials. Second, those studies employing three presentation-recall trials use the same spatial arrangement of objects thereby permitting some learning of the object-location bindings. Here, not only is the extent of learning unassessed but also it is unclear whether this learning would be expected to weaken or strengthen possible glucose effects.

Given the currently important role that the hippocampus plays in understanding glucose-related enhancements of episodic memory, the present study evaluates the influence of glucose on the hippocampal-dependent object-location memory task. To enable a reasonably broad initial evaluation, we incorporated several changes to the basic paradigm. First, multiple trials, each trial using a new spatial arrangement of objects, are given to provide a more stable estimate of memory. Second, manipulating the number of objects to remember allows an examination of working memory load and this addresses issues pertinent to the influence of task complexity on glucose-related changes. Third, using a larger (9 × 9) grid, coupled with a maximum of ten object-locations to remember, ensures that on each trial not all potential locations contain an object. Fourth, two versions of the object-location task are deployed for evaluating the generality of any effects found. In one version, the objects are common words, with their rich semantic associations. In the other version, the objects are relatively simple geometrical shapes. Finally, the free-recall data collected enables separate evaluations of memory for the objects, memory for the locations, and their joint contribution to successful object-location binding.

## Methods

### Participants

Thirty-two participants completed a single-session study lasting about 60 min. No participants had diabetes or phenylketonuria, and all were fluent in English and had normal or corrected vision. Participants were required to fast from midnight the previous night and only drink water prior to attending the morning session to ensure their blood glucose was at fasting levels. The University Research Ethics Committee approved the study, and all participants gave written informed consent prior to their participation in the study. At the end of the study, participants were asked to confirm their consent for the data to be used. Based on our study design, power calculations showed that a total of 32 participants were needed to detect (*p* = 0.05) a medium glucose effect (*d* = 0.50, *η*_p_^2^ = 0.06) with 95 % power (Faul 2007). However, data from one participant (placebo group) was excluded because they failed to confirm consent for their data to be used, leaving a sample size of 31 participants (see Table 1).

**Table 1:** Basic demographic information, blood glucose values, within-session timings, and overall performance on the object-location binding task (±SE)

	Glucose (*n* = 16)	Placebo (*n* = 15)	
Female/Male ratio	11/5	11/4	
Age (years)	22.5 (1.5)	26.5 (4.0)	
Body mass index	21.6 (0.6)	23.2 (0.9)	
Self-reported mood			
Pre-session stress	4.9 (1.1)	2.9 (1.1)	
Post-session stress	4.0 (0.8)	4.6 (0.8)	
Pre-session arousal	5.1 (0.9)	5.9 (0.9)	
Post-session arousal	6.8 (1.0)	7.1 (1.0)	
Blood glucose (mmol/L)			
Pre-session	5.16 (0.14)	4.99 (0.14)	
Mid-session	7.84 (0.23)	5.15 (0.24)	**
Post-session	7.81 (0.30)	5.11 (0.31)	**
Within-session timings (min)			
Pre- to mid-session	31.31 (1.43)	33.13 (1.47)	
Mid- to post-session	20.81 (0.92)	20.80 (0.95)	
Average session length	52.13 (2.01)	53.93 (2.07)	
Overall task performance			
Object memory (%)	67.38 (1.54)	63.06 (2.68)	
Location memory (%)	30.35 (1.29)	25.76 (1.54)	*
Object-location binding memory (%)	20.83 (1.15)	16.25 (1.00)	**
Retrieval time (s)	27.30 (1.72)	29.31 (2.11)	
Number of invalid location errors	1.96 (0.13)	1.97 (0.22)	
Number of location swap errors	0.70 (0.05)	0.68 (0.06)	
Mean conditional probability	0.47 (0.02)	0.43 (0.02)	#

Glucose effects: #*p* = 0.056; *p < 0.05; **p < 0.01

### Procedure

Participants arrived for testing at about 09:30, re-read the recruitment information, had any questions answered, provided informed consent, and the exp26rimenter administered the drink (glucose or placebo) according to a predetermined random order. A maximum of three participants were tested on each session. The drinks comprised 300 ml of water mixed with 30 ml of “no added sugar” orange and lemon squash. The glucose drink contained 30 g glucose (114 kcal or 477 kJ), and the placebo drink contained 45 mg saccharin (1.8 kcal or 7.8 kJ). Earlier work has shown that this results in a similar “mouth feel” and sweetness for the two drinks (e.g. Meikle et al. 2004). Following consent, participants had their blood glucose measured (pre-session) using the OneTouch Ultra blood glucose monitoring system (donated by Johnson & Johnson Company) and then received their allocated drink (glucose or placebo). While they waited 10 min to allow blood glucose levels to rise (Meikle et al. 2004; Stollery and Christian 2013), demographic information was collected (e.g. age, sex, BMI, fasting compliance) and they completed a stress-arousal checklist (Mackay et al. 1978) to evaluate whether stress or arousal changes mediate the effect of glucose on performance (see Meikle et al. 2004; Smith et al. 2011). They then began their first version of the object-location binding task (either the shape or word version). After finishing that version (about 20 min), there was a brief rest break during which a second (mid-session) blood glucose measure was taken. The participants then began the other version of the object-location binding task. After finishing the task, participants completed the stress-arousal checklist, made a forced choice decision about the drink they believed they had consumed, and the final blood glucose measure taken. Finally, they were thanked, received debriefing information, and reconfirmed their consent.

### The object-location binding task

**Fig. 1:**
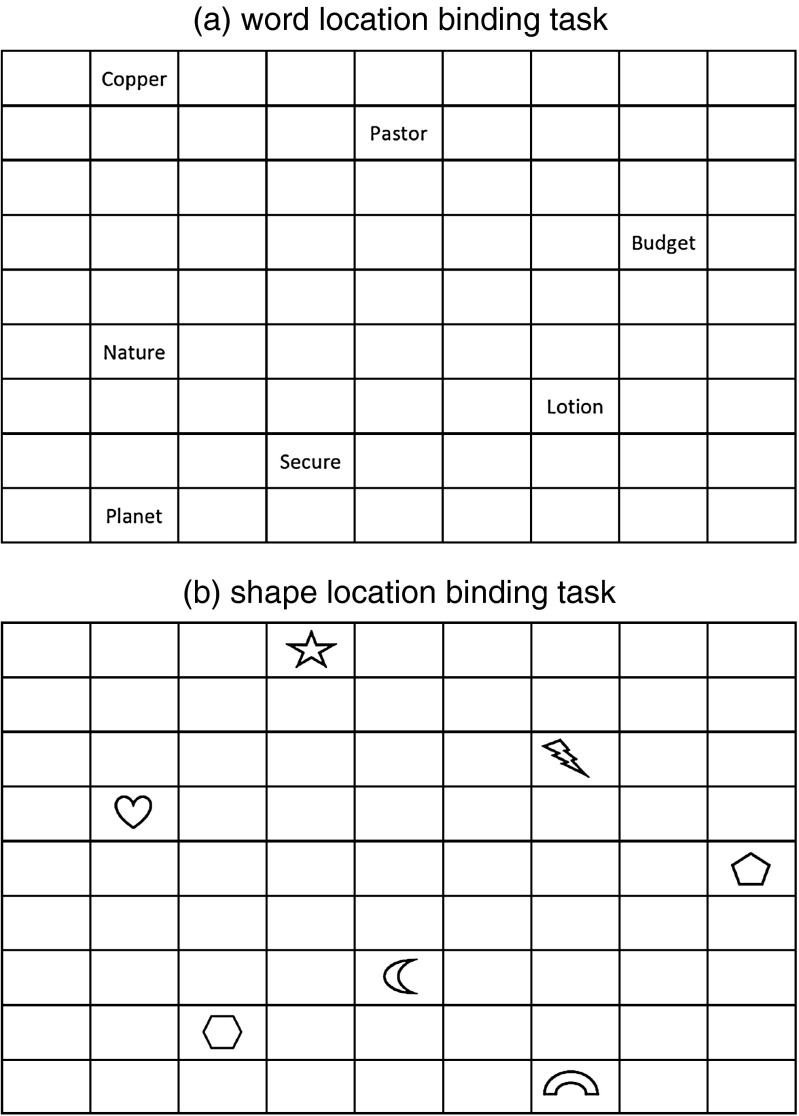
Object-location binding task with a memory load of seven objects for **a** word and **b** shape versions

Two versions of a computerized object-location binding task were used: one version used words as objects and the other used shapes (see Fig. 1). In both versions, each presentation-recall trial followed the same procedure. For the presentation phase, an empty 9 × 9 matrix was displayed continuously on a 43-cm monitor. The four corners of the 9 × 9 matrix were not used to display objects, leaving 77 possible display locations. Each trial began with the simultaneous presentation of several objects (memory load) at randomly selected matrix locations (each 25 × 15 mm) with instructions to remember the objects and their location. Objects were centred in their matrix location, and participants were free to position themselves in front of the monitor at a comfortable viewing distance. Four memory loads were used (i.e. 3, 5, 7, and 10 objects), and the total presentation time was adjusted for the number of objects displayed: 2 s plus 1.5 s per object (i.e. 6.5, 9.5, 12.5, and 17.0 s respectively across the four memory loads).

Immediately after the objects disappeared the recall phase began. For each version, participants were provided with a 24-page booklet, placed at the side of the monitor, and each page contained an empty 9 × 9 matrix. Using this empty matrix, participants were asked to write down all the objects they saw in their remembered locations, as quickly and accurately as possible. If the participant could recall an object but they were uncertain about the object’s exact location, they were asked to write the object at the location that represented “their best guess”. If a participant could recall the location of an object, but they could not retrieve any information about the object’s identity, then they simply wrote an X at the remembered location in the blank 9 × 9 matrix. After completing this timed free-recall phase, participants turned over to the next page of the booklet and initiated the next trial by pressing the space bar on a standard keyboard. The next presentation-recall trial began after 2 s. No feedback was provided.

The order of the word and shape versions of the task was counterbalanced across participants and each version lasted about 20 min. The task performance was assessed using five main indicators: correctly recalled objects, correctly recalled locations, correctly recalled objects recalled in the correct location, the time taken to recall this information, and placement errors. All performance measures were averaged across the six replications employed (see next paragraph).

Each version of the object-location binding task comprised 24 presentation-recall trials arranged as six blocks, each block containing four trials. The four trials within a block comprised one trial at each of the four memory loads (i.e. 3, 5, 7, and 10 objects) in random order. The six blocks were replications of the memory load factor and blocks followed each other without a break. For the word version, the stimuli comprised a pool of 150 words created using the MRC psycholinguistic database (Fearnley 1997). All words were six letters long, contained between 1 and 3 syllables (2.0 ± 0.34), and had mid-range values for imagability (444 ± 8.0), concreteness (433 ± 9.9), meaningfulness (397 ± 8.0), and Thorndike-Lorge written frequency (130 ± 13.5); all values shown are mean ± SE. Words were displayed in Courier 14 font and only used once. Each participant saw a different random selection from the word pool across the 24 presentation-recall trials. For the shape version, the stimuli comprised 25 geometric forms (e.g. triangle, circle, heart, and hexagon). On each trial, shapes were randomly selected such that each shape was only used once within the set of four trials that defined a replication block. The 25 shapes were reused on each of the six blocks.

### Statistical analysis

The main design for the cognitive analysis is a three-factor mixed ANOVA, with drink (glucose or placebo) as the unrelated factor and object type (word vs. shape) and memory load (3, 5, 7, and 10) as the two related factors. The partial eta-square (*η*_p_^2^) effect size for glucose is cited (0.01 ≅ small, 0.06 ≅ medium, 0.15 ≅ large) with the *F*-ratio and the Cohen’s *d* effect size is also provided for selected comparisons. When sphericity violations occur, the Huynh-Feldt corrected *p* values are reported, but the original degrees of freedom are cited for readability. The Tukey (HSD) test is used for post hoc pair-wise comparisons.

## Results

The results from the study are presented in four main sections. The first considers evidence relating to drink detection, group matching, blood glucose changes, and the assessment of stress and arousal. In the second, basic performance measures from the object-location task are reported (e.g. item memory). The third presents a conditional probability analysis to examine the relative importance of object and location memory to successful object-location binding. Finally, the influence of blood glucose measures on object-location performance is considered.

### Drink detection, blood glucose, and mood

Based on the forced-choice decision participants made at the end of their session, participants were unable to identify the drink they received (χ^2^(1, *N* = 31) = 0.32, *p* = 0.570); although the majority (84 %) believed they had consumed glucose. As shown in Table 1, the glucose and placebo groups did not differ in age (*t*(29) = 0.96, *p* = 0.345), body mass index (*t*(29) = 1.53, *p* = 0.137), or in the distribution of male/female participants (*χ*^2^(1) = 0.079, *p* = 0.779). A series of Pearson correlations between the demographic, mood, and object-location memory performance showed no significant correlations, indicating that the performance was not related to variations in age, BMI, or mood.

#### Blood glucose changes and timing

Blood glucose levels were analysed using a two-factor mixed ANOVA with drink (glucose, placebo) as the unrelated factor and time (pre-session, mid-session, post-session) as the related factor. There were main effects of drink (*F*(1,29) = 45.6, *p* < 0.001), time (*F*(1,29) = 47.0, *p* < 0.001) and a drink × time interaction (*F*(2,58) = 38.3, *p* < 0.001; MSE = 0.434). Post hoc analysis showed no pre-session differences between the glucose and placebo groups, no change in blood glucose across time for the placebo group but raised levels for the glucose group at mid-session (*p* < 0.01) that remained constant until the end of the session (see Table 1).

As the object-location task was self-paced, the relative timing of the mid- and post-session glucose measurements was evaluated (see Table 1). This showed the mid-session measurement occurred about 32 min after the pre-session measurement, with the post-session measurement about 21 min later, giving an average session duration of 53 min (SD = 8 min). The longer average interval from pre- to mid-session was due to the 10-min waiting period following glucose ingestion prior to commencing the object-location task. Analysis of the timings showed no difference in average session duration as a function of drink (*F*(1,29) = 0.393, *p* = 0.535), and there were no differences in the relative timing of the mid- and post-session timings for the glucose and placebo groups (*F*(1,29) = 0.937, *p* = 0.341).

#### Self-reported mood

Subjective evaluations of stress and arousal were analysed using a two-factor mixed ANOVA with drink (glucose, placebo) as the unrelated factor and time (pre-session, post-session) as the related factor (see Table 1). There was no evidence that glucose consumption influences stress or arousal. For arousal, there was no main effects of drink (*F*(1,29) = 0.286, *p* = 0.587), a tendency for higher arousal at the end of the session (5.5 vs. 6.9; *F*(1,29) = 3.43, *p* = 0.070), and no interaction (*F*(1,29) = 0.132, *p* = 0.719). For stress, there were no effects of drink (*F*(1,29) = 0.451, *p* = 0.507), time (*F*(1,29) = 0.271, *p* = 0.606), and no time × drink interaction (*F*(1,29) = 3.06, *p* = 0.091).

### Object-location memory task

As shown in Table 2, task performance was assessed using five primary indicators: the percentage of objects correctly recalled (object memory), the percentage of locations correctly recalled (location memory), the percentage of objects correctly recalled in the correct location (object-location binding memory), the time taken to place the recalled objects in their remembered locations (retrieval time), and placement errors. Table 1 also summarizes overall object-location performance, for each drink condition, on the different performance measures analysed.

**Table 2:** Descriptive statistics for the primary outcome measures in the object-location memory task (± SE)

	Glucose		Placebo	
	Words	Shapes	Words	Shapes
Object memory (%)				
3 objects	96.9 (2.5)	85.8 (2.8)	91.9 (2.6)	83.3 (2.9)
5 objects	79.4 (3.6)	69.8 (3.7)	71.1 (3.7)	68.0 (3.8)
7 objects	58.2 (3.5)	58.0 (3.6)	56.0 (3.6)	52.2 (3.8)
10 objects	46.5 (2.6)	44.6 (2.6)	40.9 (2.7)	41.2 (2.7)
Location memory (%)				
3 objects	50.0 (3.9)	26.0 (2.7)	40.4 (4.1)	23.4 (2.8)
5 objects	34.6 (3.3)	24.8 (2.1)	28.0 (3.4)	22.9 (2.2)
7 objects	30.2 (2.9)	22.9 (2.2)	28.8 (3.0)	19.5 (2.3)
10 objects	32.8 (2.6)	22.5 (1.6)	25.2 (2.7)	19.9 (1.6)
Object-location binding memory (%)				
3 objects	46.2 (3.7)	21.2 (2.4)	37.0 (3.9)	19.6 (2.5)
5 objects	27.1 (2.8)	15.4 (2.2)	16.7 (2.9)	14.4 (2.3)
7 objects	18.3 (2.4)	12.4 (1.7)	15.7 (2.5)	08.7 (1.8)
10 objects	17.8 (2.2)	08.3 (1.1)	10.8 (2.2)	07.1 (1.2)
Retrieval time (s)				
3 objects	16.7 (1.2)	18.0 (1.4)	16.9 (1.3)	17.9 (1.4)
5 objects	26.1 (2.5)	25.9 (2.1)	28.1 (2.5)	27.2 (2.2)
7 objects	29.9 (2.4)	31.9 (2.8)	33.2 (2.5)	33.4 (2.9)
10 objects	34.5 (2.6)	35.5 (2.9)	38.2 (2.6)	39.5 (3.0)

Initially, a check was made for changes in object memory across the six blocks of trials. This was relevant because although each block contained unique words, the 25 shapes were reused in each block. There was no evidence that object memory varied across the six blocks (*F*(5,150) = 0.276, *p* = 0.881); there was no overall advantage for recalling words or shapes (*F*(1,30) = 2.80, *p* = 0.105) and no object type × block interaction (*F*(5,150) = 0.862, *p* = 0.476). Thus, there was no evidence for either learning or a build-up of proactive inhibition across the six blocks and the accuracy of recalling the word and shape stimuli was comparable.

#### Object memory

The main effect of drink was not significant (*F*(1,29) = 2.02, *p* = 0.166; *η*_p_^2^ = 0.065) but did show a medium-size advantage for glucose (67 vs. 63 %; *d* = 0.511). Moreover, drink did not interact with object type (*F*(1,29) = 0.192, *p* = 0.664), memory load (*F*(3,87) = 0.065, *p* = 0.978), or the object type × memory load interaction (*F*(3,87) = 1.02, *p* = 0.390). Object memory declined progressively with increasing memory load (*F*(3,67) = 335.2, *p* < 0.001, *d* = 6.11), and the main effect of object type (*F*(1,29) = 4.92, *p* = 0.035) interacted with memory load (*F*(3,87) = 3.98, *p* = 0.010; MSE = 67.37).

Post hoc analysis localized the source of the interaction to the two smallest memory loads, where words were better recalled than shapes (three objects, *p* < 0.01; five objects, *p* = 0.05). The recall of words and shapes did not differ for seven and ten objects. Both words and shapes showed a progressive decline in accuracy with increases in memory load (all *p* < 0.01).

#### Location memory

Those consuming glucose were more accurate in recalling the location of objects (30 vs. 26 %; *F*(1,29) = 5.28, *p* = 0.029; *η*_p_^2^ = 0.154; *d* = 0.826), but drink did not interact with object type (*F*(1,29) = 1.01, *p* = 0.323), memory load (*F*(3,87) = 0.162, *p* = 0.907), or the object type × memory load interaction (*F*(3,87) = 0.384, *p* = 0.752) indicating a general benefit of glucose in accurately recalling locations. Location memory was better for words than shapes (33 vs. 23 %; *F*(1,29) = 29.8, *p* < 0.001), and the main effect of memory load (*F*(3,87) = 14.1, *p* < 0.001) interacted with object type (*F*(3,87) = 8.32, *p* < 0.001; MSE = 71.95).

Post hoc analysis of the interaction localized the source to the three-word memory load. Memory for the location of words was better for three compared to five words (45 vs. 31 %, *p* < 0.01) and thereafter remained constant at 29 %. For shapes, location memory did not vary with memory load (25, 24, 21, and 21 %, respectively). Thus, location memory was constant across memory loads, with the exception of the three-word condition where there was better location memory. The better location memory for words was present at all memory loads (three objects, *p* < 0.01, and all other memory loads, *p* < 0.05).

#### Object-location binding memory

Those receiving glucose showed better object-location binding (21 vs. 16 %; *F*(1,29) = 8.94, *p* = 0.006; *η*_p_^2^ = 0.326; *d* = 1.075), and this benefit did not interact with object type (*F*(1,29) = 2.32, *p* = 0.139), memory load (*F*(3,87) = 0.30, *p* = 0.787), or the object type × memory load interaction (*F*(3,87) = 1.22, *p* = 0.305), indicating a general advantage of glucose on binding effectiveness. In addition, word-location binding was more successful than shape-location binding (*F*(1,29) = 33.2, *p* < 0.001); binding became generally less efficient with increasing memory load (*F*(3,87) = 68.7, *p* < 0.001); and there was an object type × memory load interaction (*F*(3,87) = 12.2, *p* < 0.001; MSE = 78.17).

Post hoc analysis of the interaction localized the source to the smallest memory load. When three objects were shown, word-location binding was better than shape-location binding (42 vs. 20 %, *p* < 0.01). When five objects were presented, word-location binding declined by 20 % (*p* < 0.01); shape-location binding showed a marginal decline (5.5 %, critical difference = 7.02 % for *p* < 0.05), with word-location binding remaining superior. As memory load increased from five to ten objects, word-location binding remained superior to shape-location binding (*F*(1,29) = 16.0, *p* < 0.001, *d* = 1.03) and binding effectiveness declined (*F*(2,58) = 19.3, *p* < 0.001, *d* = 1.21) at the same rate for words and shapes (*F*(2,58) = 0.02, *p* = 0.979).

#### Retrieval time

Retrieval times did not differ as a function of drink (*F*(1,29) = 0.55, *p* = 0.463) and object type (*F*(1,29) = 0.30, *p* = 0.590) but increased with memory load (*F*(3,87) = 144.7, *p* < 0.001). No other effects approached significance and, in particular, drink did not interact with object type (*F*(1,29) = 0.05, *p* = 0.820), memory load (*F*(3,87) = 1.29, *p* = 0.283), or the object type × memory load (*F*(3,87) = 0.31, *p* = 0.816) interaction. Post hoc analysis simply showed that the time taken to recall objects increased with the number of objects to recall (17, 27, 32, and 37 s respectively; all *p* < 0.01).

**Fig. 2:**
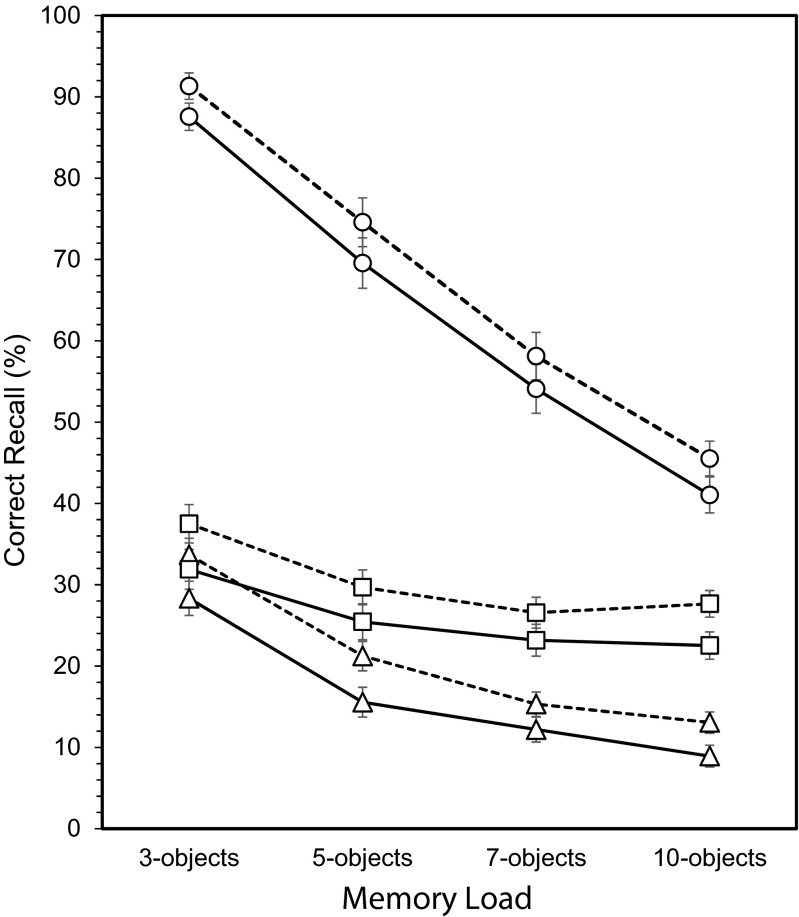
Object, location, and binding memory as a function of memory load and glucose consumption (±SE). *Dashed line* glucose, *solid line* placebo. ○ item memory, □ location memory, Δ object-location binding memory

In summary, as shown in Table 1, those receiving glucose showed a particularly strong facilitative effect on object-location binding (*d* = 1.075) and location memory (*d* = 0.826), with only weak evidence for improved object memory (*d* = 0.511). Increasing the number of objects to remember had a pronounced and detrimental impact on object memory and a smaller, but reliable, decrement in object-location binding. Except for the three-word condition, location memory did not vary with memory load. Although location memory for words was better than for shapes and word-location binding was generally more successful than shape-location binding, none of these effects varied with the consumption of glucose. Thus, the benefits of glucose on object-location binding and location memory did not vary according to task difficulty as assessed by changes in memory load or the type of object to remember. The invariance of the glucose effect across the different memory loads for object memory, location memory, and object-location binding is illustrated in Fig. 2.

#### Object-location errors

Given the superior recall of objects compared to locations, there are two important kinds of error to consider for the misplacement of correctly recalled objects. Participants can place a recalled object at either an unused location (invalid location error) or a valid location that is invalid for that object (location swap error). While both errors represent failures in selecting the correct location of a valid object, the invalid location error has no subsequent impact on task performance because the selected location did not contain an object. In contrast, location swap errors entail placing an object at the location of another object. It follows that subsequently recalling the object originally at that location must also give rise to an error because a different object now occupies the valid location. To explore the influence of glucose on these two kinds of error, a four-factor mixed ANOVA with error type (invalid location, location swap), object type (word, shape), and memory load (3, 5, 7, and 10) as the related factors and drink (glucose, placebo) as the unrelated factor was conducted on the number of errors made.

The analysis shows no main effect of drink (*F*(1,29) = .001, *p* = 0.986) and none of the interactions with drink approach significance. The most common error was placing the recalled object at an invalid location (1.97 vs. 0.69, *F*(1,29) = 72.0, *p* < 0.001), and more errors were made when the object was a shape (1.41 vs. 1.25, *F*(1,29) = 5.10, *p* = 0.032). The general increase in errors with memory load (*F*(3,87) = 66.4, *p* < 0.001) further interacted with (a) error type (*F*(3,87) = 23.6, *p* < 0.001; MSE = 0.470) and (b) object type (*F*(3,87) = 2.94, *p* = 0.037; MSE = 0.133). Neither the error type × object type × memory load (*F*(3,87) = 1.83, *p* = 0.153) or its interaction with drink (*F*(3,87) = 0.38, *p* = 0.738) was significant.

**Fig. 3:**
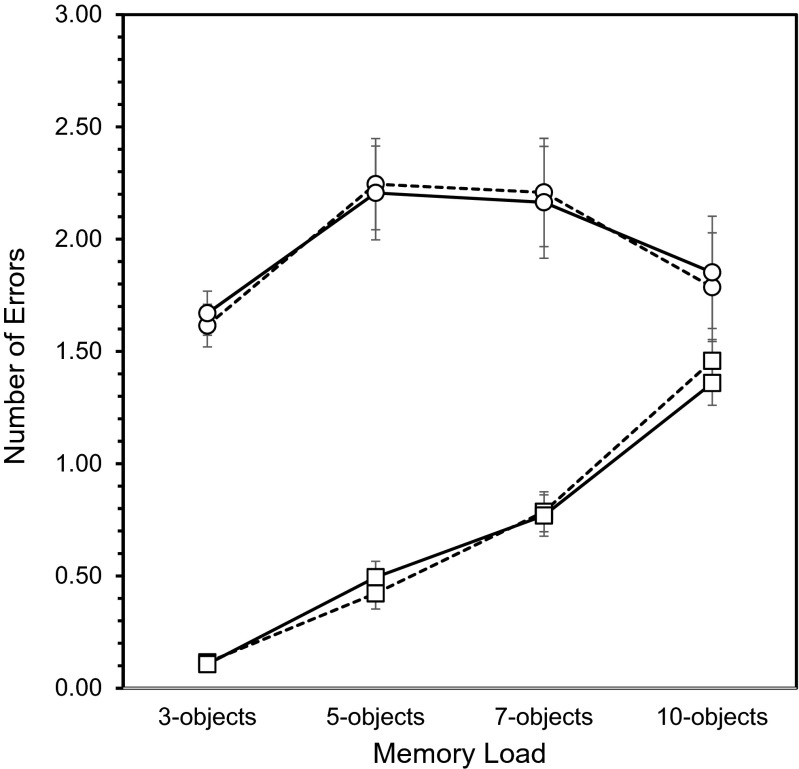
Object-location binding errors (±SE) for the glucose and placebo groups. *Dashed line* glucose, *solid line* placebo. ○ invalid location error, □ location swap error

As shown in Fig. 3, post hoc analysis of the error type × memory load interaction showed invalid location errors exhibited a shallow inverted U-shaped effect across memory load: errors increased from 3 to 5 objects (*p* < 0.01), showed no change from 5 to 7 objects, and then showed a small decline from 7 to 10 objects (*p* = 0.050). In contrast, the number of location swap errors increased progressively across memory load (*p* < 0.01). Invalid location errors were more common than location swap errors at each memory load.

For the object type × memory load interaction, post hoc analysis localized the source to the highest memory load. Words and shapes produced equivalent binding errors for 3, 5, and 7 objects, but shapes showed more binding errors for 10 objects (*p* < 0.01).

In conclusion, the most common binding error was placing recalled objects at an invalid location, words showed the same kinds of binding errors as shapes (except at the highest memory load), and the two kinds of binding error showed distinct influences of memory load. However, there were no effects of glucose on the incidence or type of error made.

### Conditional probability analysis

The above analyses indicated that glucose preferentially improves location memory and object-location binding memory but did not influence the nature and type of binding errors made. Next, we examine the relative importance of object identity and object location in this successful binding using a conditional probability analysis. This analysis examines the probability of one event occurring, given that another event has already occurred. In the context of the current task, when binding two attributes of an object together, the probability of recalling one attribute (e.g. the object) can be conditional on the ability to recall the other attribute (e.g. the location). Thus, there are two conditional probabilities of interest. The first is the probability of recalling the object, given that the location has been successfully recalled–given by *p*(O|L). The second is the probability of recalling the location, given that the object has been successfully recalled–given by *p*(L|O). As these two conditional probabilities need not be symmetric, especially in light to the special status of location memory (e.g. Caprio et al. 2010), the following analyses examine the impact of glucose on both conditional probabilities.

A four-factor mixed ANOVA with drink (glucose, placebo) as the unrelated factor and conditional probability type (*p*(O|L), *p*(L|O)), object type (word, shape), and memory load (3, 5, 7, and 10) as the three related factors was conducted on the conditional probabilities (see Table 3). Average conditional probabilities tended to be higher for those receiving glucose (0.471 vs. 0.425; *F*(1,29) = 3.97, *p* = 0.056; *η*_p_^2^ = 0.120; *d* = 0.730). Although the glucose effect size was numerically greater for *p*(L|O) than *p*(O|L) (*d* = 0.654 vs. 0.508), there was no evidence that drink interacted with conditional probability type (*F*(1,29) = 0.003, *p* = 0.960), object type (*F*(1,29) = 2.59, *p* = 0.118), and memory load (*F*(3,87) = 0.885, *p* = 0.452); and none of the glucose interactions approached significance.

**Table 3:** Descriptive statistics for the conditional probabilities in the object-location memory task (± SE)

Conditional probability	Object type	Memory load	Glucose		Placebo	
*p*(O|L)^a^	Words	3 objects	0.953	(0.030)	0.919	(0.031)
		5 objects	0.799	(0.050)	0.594	(0.052)
		7 objects	0.584	(0.043)	0.590	(0.045)
		10 objects	0.527	(0.045)	0.428	(0.047)
	Shapes	3 objects	0.832	(0.039)	0.862	(0.041)
		5 objects	0.595	(0.060)	0.596	(0.062)
		7 objects	0.498	(0.050)	0.463	(0.051)
		10 objects	0.369	(0.037)	0.333	(0.038)
*p*(L|O)^b^	Words	3 objects	0.477	(0.038)	0.401	(0.039)
		5 objects	0.339	(0.032)	0.237	(0.033)
		7 objects	0.309	(0.036)	0.294	(0.037)
		10 objects	0.368	(0.042)	0.272	(0.043)
	Shapes	3 objects	0.249	(0.031)	0.244	(0.032)
		5 objects	0.224	(0.033)	0.219	(0.034)
		7 objects	0.210	(0.030)	0.180	(0.031)
		10 objects	0.197	(0.028)	0.172	(0.029)

^a^Probability of recalling the object (given the location was correct)

^b^Probability of recalling the location (given the object was correct)

The main effect of conditional probability type (*F*(1,29) = 381, *p* < 0.001; *η*_p_^2^ = 0.929; *d* = 4.439) showed that the probabilities were asymmetric: correctly recalling a location more effectively predicted the probability of recalling the object at that location (*p*(O|L) = 0.621) than correctly recalling an object predicted the probability of recalling the location of that object (*p*(L|O) = 0.275). Conditional probabilities were also generally higher for words than shapes (0.506 vs. 0.390; *F*(1,29) = 35.5, *p* < 0.001, *d* = 1.344), and the main effect of memory load (*F*(3,87) = 58.7, *p* < 0.001, *d* = 3.518) entered into two interactions: a conditional probability type × memory load interaction (*F*(3, 87) = 76.8, *p* < 0.001) which further interacted with object type (*F*(3, 87) = 3.40, *p* = 0.021).

**Fig. 4:**
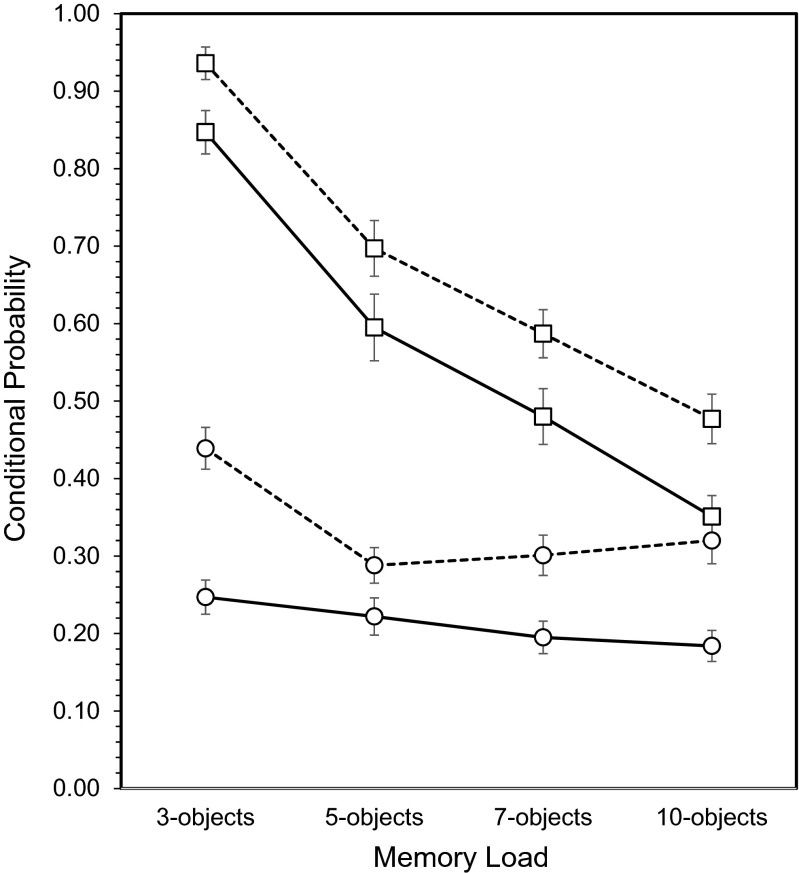
Conditional probabilities as a function of object type and memory load (± SE). □ *p*(O|L), ○ *p*(L|О). *Dashed line* words, *solid line* shapes

Follow-up analysis of the three-way interaction showed the basic memory load × object type interaction was restricted to *p*(L|O): the probability of recalling the correct location, conditional on successful object recall. Specifically, as shown in Fig. 4, *p*(L|O) was higher for words than shapes (*F*(1,29) = 27.6, *p* < 0.001), and the main effect of memory load (*F*(3,87) = 9.79, *p* < 0.001) interacted with object type (*F*(3,87) = 3.72, *p* = 0.014; MSE = 0.012). In contrast, although *p*(O|L) was also higher for words (*F*(1,29) = 18.0, *p* < 0.001) and declined with memory load (*F*(3,87) = 87.3, *p* < 0.001), the object type × memory load interaction did not approach significance (*F*(3,87) = 0.155, *p* = 0.926).

Post hoc analysis showed that the object type × memory load interaction for *p*(L|O) was due to the three-word condition. Given successful word recall, the probability of recalling the location of that word declined from three words to five words (*p* < 0.01) and then remained constant. For shapes, the probability of recalling the location of a correctly recalled shape did not vary with memory load. Furthermore, for memory loads of five and more objects, *p*(L|O) was higher for words (*F*(1,29) = 17.6, *p* < 0.001), showed no effect of memory load (*F*(2,58) = 0.061, *p* = 0.941), and no object type × memory load interaction (*F*(2,58) = 1.73, *p* = 0.186).

#### Correction for guessing

The possibility that *p*(L|O) was overestimated due to guessing needs to be considered. On some trials, an object (e.g. a star) can be correctly recalled, but there is uncertainty about the object’s exact location. In these situations, the participant’s guess can place the recalled object at the correct location, the location of another object (location swap error), or at an unused location (invalid location error). In the current study, invalid location errors were the dominant error, although location swap errors are an established binding error (see Pertzov et al. 2013; Postma and De Haan 1996; Watson et al. 2013). The problem with estimating *p*(L|O) arises when the guess places the object at one of the valid locations because then the observed proportion of location correct trials will overestimate the “true” value of *p*(L|O). One solution to this, proposed by Dent and Smyth (2005), assumes that when uncertain of a recalled object’s location, a random choice is made from all the available locations. Explicitly, with 77 possible object locations, participants can correctly guess the location of a correctly recalled object with a probability of 0.0129 (i.e. 1 in 77). According to Dent and Smyth (2005), p. 122 the true *p*(L|O) is therefore given by the following equation: 1 + ((*n*(*p*(L|O) − 1))/(n − 1)), where *n* is the total number of locations to guess from (i.e. 77) and *p*(L|O) is the uncorrected conditional probability. With this assumption, it was possible to correct for location guessing when an object was successfully recalled. This account, while not accounting explicitly for location swap errors, does seem reasonably appropriate given the small number of locations used (max = 10), the large number of available locations to select from (*n* = 77), and the low incidence of location swap errors (mean = 0.69). While the application of this guessing correction reduced the average value of *p*(L|O) from 0.275 to 0.269 (*p* < 0.001), a reanalysis of the data showed no changes in the pattern or significance of the findings reported earlier.

In summary, correctly recalling the location of an object more effectively supported recalling the identity of the object at that location than vice versa (i.e. *p*(O|L) > *p*(L|O)), and both conditional probabilities were higher for words than shapes. When participants successfully recalled locations, the probability of recalling the object at that location declined as the number of objects to remember increased. In contrast, when the participant successfully recalled an object, the probability of recalling the location of that object did not vary with memory load, except for when three words were presented when it was higher. Of particular importance, glucose tended to improve both conditional probabilities, and this indicated that it was equally effective in facilitating object recall (given correct location recall) and location recall (given correct object recall). Furthermore, this influence of glucose was not dependent on whether the objects were words or shapes or difficulty as indexed by the number of objects to remember.

### Blood glucose and object-location memory

Our final analyses consider the influence of blood glucose measures on object-location memory. The trapezoid procedure described by Pruessner et al. (2003) was used to estimate two measures of regulation efficiency based on the area under the curve (AUC): AUC with respect to ground (AUC_G_ range 278–524 min mmol/L) and AUC with respect to increase (AUC_I_ range 37–240 min mmol/L). Using the following abbreviations–blood glucose at baseline (BG_0_), mid-session (BG_1_) and post-session (BG_2_), and the time interval (in minutes) between baseline and mid-session (*T*_01_), mid-session and post-session (*T*_12_), and baseline and post-session (*T*_02_)–the two regulation indices are given by the following equations:

$$ \begin{array}{c}\kern1em {\mathrm{AUC}}_{\mathrm{G}}=\left[\left({\mathrm{BG}}_0+{\mathrm{BG}}_1\right)\times {T}_{01}\Big)/2\right]+\left[\left({\mathrm{BG}}_2+{\mathrm{BG}}_3\right)\times {T}_{12}\Big)/2\right]\kern1em \\ {}\kern1em {\mathrm{AUC}}_{\mathrm{I}}={\mathrm{AUC}}_{\mathrm{G}}\hbox{-} \left({\mathrm{BG}}_0\times {T}_{02}\right).\kern1em \end{array} $$

Several authors point out that each measure provides unique information (e.g. Le Floch et al. 1990; Owen et al. 2013; Pruessner et al. 2003; Sünram-Lea et al. 2011), with AUC_G_ taking into account fasting glucose levels in the total circulating glucose and AUC_I_ the sensitivity of the system to the glucose load, irrespective of baseline glucose levels. For both measures, higher values are taken to indicate poorer regulation efficiency.

Table 4 displays the correlations between task performance and various measures of glucose, including AUC_G_ and AUC_I_. For the placebo group, the calculated values of AUC_G_ (range 207–403 min mmol/L) and AUC_I_ (range −14 to 29 min mmol/L) were also incorporated. Given the large difference between the two types of conditional probability, all three measures of conditional probability are shown (i.e. *p*(O|L), *p*(L|O), and *p*(mean)) as are both forms of binding error. For completeness, correlations with matching variables (e.g. age, BMI) are also shown.

**Table 4:** Pearson correlations (two-tailed) between object-location memory, selected demographics, and blood glucose measures (*n* = 31)

					Conditional probability			Error type	
	Object memory	Location memory	Binding memory	Retrieval Time	*p*(O|L)	*p*(L|O)	*p*(mean)	Invalid location	Location swap
Sex^a^	0.174	0.161	0.118	−0.060	−0.038	0.039	−0.005	0.092	0.105
Age (years)	−0.211	−0.210	−0.165	0.038	−0.078	−0.094	−0.104	−0.071	−0.196
BMI (kg/m^2^)	−0.012	−0.328	−0.280	−0.047	−0.120	−0.301	−0.245	0.174	−0.185
Self-reported stress	0.087	0.214	0.187	−0.325	0.063	−0.003	0.060	−0.031	0.030
Self-reported arousal	0.076	−0.240	−0.221	−0.017	−0.144	−0.433	−0.317	0.191	0.021
Task order ^a^	−0.158	−0.190	−0.136	−0.078	0.002	−0.082	−0.043	−0.034	−0.099
Glucose belief ^a^	0.073	−0.150	−0.086	0.009	0.178	−0.030	0.107	0.090	−0.152
Blood glucose: baseline	−0.156	0.103	0.101	−0121	−0.054	0.149	0.043	−0.236	0.076
Blood glucose: average^b^	0.117	0.453**	0.571**	−0.132	0.317	0.475**	0.475**	−0.160	−0.016
Actual AUC_G_	−0.058	0.425*	0.537**	0.469**	0.331	0.566**	0.534**	−0.265	−0.035
Actual AUC_I_	−0.159	0.415*	0.559**	0.013	0.383*	0.458**	0.511**	−0.080	−0.083

^a^Point biserial correlation

^b^Average of mid- and post-session

*p < 0.05; **p < 0.01

As Table 4 shows, demographic variables, mood, and baseline glucose levels were not correlated with any performance measure. As expected from the previous analyses, higher glucose levels were associated with better location memory, better binding memory, and higher conditional probabilities. The same pattern was seen for the two measures of regulation efficiency, with poorer glucose regulation being associated with better performance. There are two points of interest to note. First, when the two types of conditional probability were separated, the correlations were stronger for *p*(L|O) than for *p*(O|L). Specifically, for *p*(L|O) the correlations were significant, but for *p*(O|L), correlations with blood glucose (*p* = 0.082) and AUC_G_ (*p* = 0.069) only showed trends. Second, the correlation between retrieval time and AUC_G_ suggests that poorer regulators take longer to complete the retrieval phase.

**Fig. 5:**
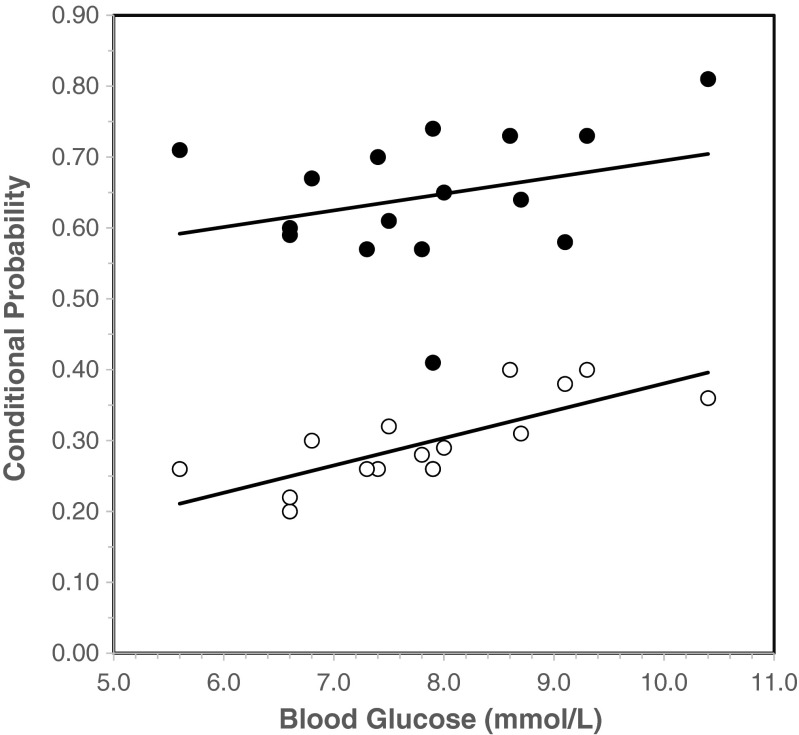
Linear regression lines showing the correlation between the two conditional probability measures and mid-session blood glucose levels within the glucose group. *● p*(О*|L*), ○ *p*(*L|*О)

To examine this further, Table 5 shows the correlations within the glucose and placebo groups. For the glucose group, higher mid-session blood glucose was associated with better location memory (*r*(14) = 0.551, *p* = 0.027), better binding memory (*r*(14) = 0.609, *p* = 0.012), and higher conditional probabilities (*r*(14) = 0.558, *p* = 0.025). Again, the correlations were stronger for *p*(L|O) than *p*(O|L), *r*(14) = 0.755, *p* = 0.001 and *r*(14) = 0.288, *p* = 0.279, respectively, and the difference between these two correlations was significant (*z* = 2.11, *p* = 0.034; see Fig. 5). Changes in blood glucose from the mid- to post-session period were not associated with any performance measure. For AUC_G_ and AUC_I_ respectively, there were no significant correlations with location memory (*r*(14) = 0.218, *p* = 0.416 and *r*(14) = 0.260, *p* = 0.331), only weak effects for binding memory (*r*(14) = 0.380, *p* = 0.146 and *r*(14) = 0.444, *p* = 0.085), but a stronger relationship with conditional probabilities (*r*(14) = 0.512, *p* = 0.043 and *r*(14) = 0.597, *p* = 0.015). Again, while the correlations were stronger for *p*(L|O) than *p*(O|L), the difference between the two correlations was not significant for either AUC_G_ (*z* = 0.704, *p* = 0.482) or AUC_I_ (*z* = 0.603, *p* = 0.547). Finally, as with the earlier analysis, higher AUC_G_ values were associated with longer retrieval times (*r*(14) = 0.611, *p* = 0.012). Interestingly, within the placebo group, only the correlation between AUC_G_ and retrieval times (*r*(13) = 0.831, *p* < 0.001) was significant.

**Table 5:** Pearson correlations (two-tailed) between object-location memory and within group measures of blood glucose

					Conditional probability			Error type	
	Object memory	Location memory	Binding memory	Retrieval time	*p*(O|L)	*p*(L|O)	*p*(mean)	Invalid location	Location swap
Glucose group (*n* = 16)									
Blood glucose: baseline	−0.166	0.214	0.075	−0.294	−0.168	0.156	−0.048	−0.348	0.310
Blood glucose: mid-session	−0.145	0.551*	0.609*	0.075	0.288	0.755**	0.558*	−0.468#	−0.047
Blood glucose: change^a^	−0.167	−0.155	−0.170	−0.289	0.016	−0.100	−0.035	−0.050	−0.033
Actual AUC_G_	−0.167	0.218	0.380	0.611*	0.362	0.542*	0.512*	−0.242	−0.227
Normalized AUC_G_	−0.234	0.425	0.453#	−0.077	0.237	0.641**	0.468#	−0.481#	−0.020
Actual AUC_I_	−0.117	0.260	0.444#	0.359	0.449#	0.594*	0.597*	−0.211	−0.324
Normalized AUC_I_	−0.146	0.332	0.470#	0.133	0.402	0.628**	0.580*	−0.295	−0.257
Placebo group (*n* = 15)									
Blood glucose: baseline	−0.275	−0.167	−0.045	0.138	0.022	0.062	0.054	−0.179	−0.232
Blood glucose: average ^b^	−0.261	−0.123	−0.058	−0.014	0.006	0.040	0.030	−0.175	−0.187
Actual AUC_G_	−0.399	0.309	0.298	0.831**	0.034	0.475	0.325	−0.435	0.103
Normalized AUC_G_	−0.322	−0.144	−0.081	0.013	−0.040	0.040	−0.003	−0.217	−0.187
Actual AUC_I_	−0.073	0.096	−0.062	−0.162	−0.150	−0.033	−0.127	−0.072	0.151
Normalized AUC_I_	−0.043	0.070	−0.059	−0.256	−0.119	−0.051	−0.116	−0.042	0.123

^a^Mid- to post-session increase

^b^Average of mid- and post-session

*p < 0.05; **p < 0.01; #*p* < 0.10

The observation that higher AUC_G_ values were correlated with longer retrieval times in both the glucose and placebo groups warrants closer inspection. The calculation of AUC_G_ incorporates the time interval between blood glucose measures (see Pruessner et al. 2003) and this interval is, in turn, determined by individual differences in the average recall time on this self-paced task. Indeed, the correlation between total session length and average recall time (*r*(29) = 0.916, *p* < 0.001) simply indicates that the time interval between blood glucose measures was longer for slower participants. As longer intervals between glucose measurements increase AUC values, there exists some confounding between AUC values and average recall time.

In an effort to disentangle individual variations in session duration from individual variations in AUC measures, an attempt was made to normalize AUC scores, operationalized by dividing an individual’s AUC score by their session length (in minutes) and then pro-rating this value to the average session length (53 min). It is important to note that there were no differences in the relative timing of blood glucose measurements for the glucose and placebo groups. We applied this normalization procedure to AUC_G_ and AUC_I_ because both incorporate the time interval between blood glucose measurements. As shown in Table 5, using these normalized measures, the correlation between AUC_G_ and recall time was no longer significant for either the glucose (*r*(14) = −0.077, *p* = 0.778) or the placebo (*r*(13) = 0.013, *p* = 0.962) group. Thus, for the placebo group, no significant associations remained between glucose measures and task performance. In contrast, for the glucose group, the other performance associations with AUC_G_ and AUC_I_ were robust to this normalization.

In summary, for the glucose group, higher measures of glucose were related to better performance on the object-location task. The most compelling of these were the positive associations with object-location binding memory and *p*(L|O). In contrast, for the placebo group, there were no significant glucose correlations with object-location performance. The initial observation that higher AUC_G_ values were associated with slower recall times in both the placebo and glucose groups was likely to result from the integration of the time between glucose measurements into AUC measures because these associations were eliminated once the AUC measures were normalized for session duration.

## Discussion

The current study examined the influence of a 30-g glucose drink on object-location memory. Participants viewed a variable number of objects (words or shapes) in different locations and immediately attempted to reproduce the objects in their remembered locations. Our basic findings indicate that glucose consumption improves the ability to remember the locations of objects and to successfully bind objects to their location. Furthermore, while increasing the number of objects to remember produces a detrimental effect on recalling the objects and on how successfully those objects were bound to locations, the beneficial influence of glucose remained stable across this difficulty manipulation.

Many researchers have noted that the binding objects to locations requires communication between the ventral (what) and dorsal (where) anatomical processing streams (Bentley and Salinas 2009; Eichenbaum et al. 2012) and on the basis of previous research on object-location memory (e.g. Crane and Milner 2005; Gilbert and Kesner 2004; Hannula and Ranganath 2008; Olson et al. 2006; Piekema et al. 2006; Postma et al. 2008), the preferential impact of glucose on binding memory is consistent with a hippocampal influence. Indeed, as the hippocampal system receives converging information from several association cortices (Shastri 2002), it is well suited to the fundamental process of binding diverse aspects of an experience into a unified episodic representation. The two principal theories of hippocampal function concern its involvement in the creation of episodic memories (e.g. Squire 1992) and its importance for spatial cognition (e.g. O'Keefe and Nadel 1978). Reconciliation of these two conceptions has been proposed (Eichenbaum 1999; Eichenbaum and Cohen Neal 2014), and the object-location task used here explicitly exploits both the episodic and spatial functions. In addition to enhancing the binding of object-location pairs, we found that glucose exerts a stronger influence on location memory than on object memory, suggesting a special role for glucose in enhancing human spatial memory. This is consistent with the body of human work on spatial cognition and the hippocampus (see Hartley et al. 2014) and parallel observations in rodents of both hippocampal contributions to spatial memory (Eichenbaum 1999; Geva-Sagiv et al. 2015; Mumby et al. 2002; Squire 1992) and improvements of spatial memory with glucose (Dash et al. 2006; McNay et al. 2000; Winocur and Gagnon 1998).

Following the creation of a bound representation comprising location and identity attributes, the retrieval of one attribute acts as a retrieval cue for the other. Although this reciprocal cueing could be symmetrical, our conditional probability analysis shows that the temporary binding created is strongly asymmetric. Specifically, remembering a location more effectively aids recalling the object at that location than remembering an object aids recalling the location of that object. This asymmetry accords well with past research showing that location has a special status in the processing of objects (e.g. Pertzov and Husain 2014; Rajsic and Wilson 2014; Roth and Franconeri 2012). The other work has shown that attending to specific locations can initiate object processing at that location (Treisman and Zhang 2006) and while the object’s location seems to be automatically encoded when attention is directed to non-spatial attributes (e.g. colour), these non-spatial attributes are not necessarily encoded when attention is simply drawn to the location (e.g. Chen 2009; Golomb et al. 2014a). Similarly, in change detection tasks, the memory for object identity is impaired by changing the locations of objects, but memory for location is unaffected by changes in object identity (Jiang et al. 2000, 2004; Poch et al. 2010). Therefore, one way of understanding this asymmetric binding strength within object-location pairs is that location provides the nucleus for the binding process. Our memory for where an object was seen is strongly bound to the memory about what object was seen there. In contrast, our memory for what the object was is only weakly bound to where it was seen. Other authors have noted the pervasiveness of such processing asymmetries in the visual system, presumably because an object must occupy a specific spatial location, whereas it is not necessary for space to contain an object (e.g. Chen 2009). It is important to note that this asymmetric binding is common to those who consumed placebo and glucose, and there is no compelling evidence that this basic asymmetry is altered by consuming glucose. Rather, glucose simply appears to strengthen the probability of gaining access to each attribute of the bound pair, given memory for the other attribute.

In connection with this asymmetry, we also found superior location memory and object-location binding, for words compared to shapes. One basis for this advantage could be the rich semantic connections of words, compared to shapes, since this creates greater contextual detail, thereby supporting retrieval. However, this advantage would also be expected to improve object memory and there is no evidence for this except at low memory loads. The most likely source is that each word is only used once, but each shape is used six times. This renders word-location bindings unique because each word had never appeared anywhere else. In contrast, shape-location bindings are not unique because when a shape is repeated, it needs to be bound to a new location. This differential mapping seems to render shape-location binding less stable (e.g. enduring bindings hindering the creation of new ones). If, as suggested above, location provides the nucleus for the binding process, then this is consistent with the observations that both types of conditional probability are lower for shapes, that location memory is poorer for shapes, and location-based errors are more common for shapes. Finally, the asymmetric cueing between object and location is preserved across both shapes and words for all memory loads, with one notable exception: the three-word condition for *p*(L|O). For this condition, memory for words, locations, and bindings are especially high and the outcome is a selective enhancement on remembering where a correctly recalled word was. Again, this points to a central role for location in the binding process, but at the lowest memory load greater accuracy concerning where a remembered word was seen (e.g. via retrieval of semantic detail). In any case, whatever processes underlie differences in shape-location and word-location binding across the different memory loads, the current study found no evidence that glucose modifies those processes.

While the evidence above indicates that glucose simply strengthens the temporary bond between location and identity attributes irrespective of the bond direction and type of object, the analysis of blood glucose variations suggests a more nuanced picture. Across all participants, baseline glucose levels were not correlated with any aspects of performance, but higher levels following glucose consumption were associated with better location memory, object-location binding memory, and higher average conditional probabilities. Critically, a separation of the two conditional probabilities indicated that higher blood glucose, and the two measures of area under the curve, tended to be more reliably correlated with the weaker of the two bonds: *p*(L|O), retrieving the location of a successfully recalled object. This pattern was only seen within the glucose group. Furthermore, this pattern is consistent with the glucose effect size being (non-significantly) stronger for *p*(L|O) than *p*(O|L): *d* = 0.654 vs. 0.508. This raises the intriguing possibility that higher glucose levels may preferentially strengthen the weaker of the two bonds within object-location pairs. That is, an effect of task complexity with glucose having a stronger impact on the more difficult component. The question therefore is how difficulty or complexity should be conceived in this situation, and to understand this we must turn to the respective roles of object and location information. The features that identify an object rarely change over time, but this is not true for the location of that object. It follows that binding an object to a location is always temporary and in normal circumstances the link needs continual updating. The relative weakness of this link may be an advantage, permitting the required flexibility for unbinding, renewing, or updating the link between the object and its current location. Knowing what an object was does not pose this updating problem. Consistent with this idea, our data shows access to what knowledge declines rapidly as memory load increases, but access to where knowledge is not influenced by memory load. For the conditional probabilities, access to objects at remembered locations is subject to this memory load effect, but access to locations from remembered objects is not. Thus, the enhancement of this weaker bond by glucose may indicate that glucose plays a preferential role in adaptably binding an object to its current location. Thus, difficulty could be broadly conceived as relating to the differential demands of flexibly updating information.

However, given the small sample sizes, it would be premature to infer too much from the different strengths of the glucose relationships with the two conditional probabilities. The main evidence suggests that glucose improves both bonds equivalently, rather than one more than another. Based on our correlational analysis, for those given glucose, we simply find that higher glucose levels are associated with better binding memory and conditional probability scores. These positive correlations may also be interpreted as reflecting the influence of glucose regulation efficiency, with poor regulators showing the most improvement. The selective strengthening of the weaker of the two bonds linking object and location, however, must remain tentative. This is not simply because only one glucose measure shows a significant difference in correlations but also because an interactive influence of glucose on the two conditional probabilities was not detected in the main analysis. Additionally, the improvement in location memory following glucose consumption is clearly independent of blood glucose variations within the glucose group.

There are a number of potential process, discussed below, that could underlie the strengthening of the bond between objects and locations. Further work is clearly required for a more complete understanding of how this strengthening occurs, but it seems likely that enhanced location memory will occupy a central role. While early models proposed that attention binds or “glues” different features of objects together (Treisman 1988), simple objects comprising intrinsic combinations of single features (e.g. blue circle) are not attention demanding, being processed automatically prior to entering the episodic buffer of working memory (Allen et al. 2012). The binding of objects to locations is also thought to occur automatically (Chen and Wyble 2015; Treisman and Zhang 2006) but appears particularly fragile because delays of several seconds can disrupt this binding, particularly with high-memory loads (Pertzov et al. 2012). This disruption is not due to failures in remembering objects, but declines in the precision of remembering locations, pointing to difficulties in maintaining bound representations (see Olson et al. 2006). Other work has shown that maintaining bindings involving location produces sustained activation of the hippocampus, whereas maintaining non-spatial bindings does not (Piekema et al. 2006). Consistent with this are the deficits in object-location memory for patients with hippocampal damage (Hampstead et al. 2011; Hannula and Ranganath 2008; Hannula et al. 2006; Yee et al. 2014), with the prefrontal cortex supporting both the encoding (Spellman et al. 2015) and the maintenance (Campo et al. 2005; Prabhakaran et al. 2000) of spatial information in working memory. Contrary to this evidence, Allen et al. (2014) report preserved colour-location binding for up to 10 s in a patient with selective loss of hippocampal tissue. However, importantly, binding fell to chance levels when assessed only a few minutes later. Thus, while it remains plausible that glucose promotes the formation of object-location bindings, the above observations suggest that their fragility lies in maintaining them and this seems the most likely source of the glucose facilitation observed.

One important function of the episodic buffer, which acts as an interface between working memory and longer-term episodic memory, is maintaining integrated memory representations (Baddeley et al. 2011). Whether the hippocampus can feasibly represent the anatomical site linking working and longer-term episodic memory remains unresolved (see Baddeley et al. 2011 for a discussion), but if glucose supports memory maintenance, then several bases for the enhanced binding are possible. Initially, it would seem sensible to establish that improved binding due to glucose is restricted to tasks with a spatial basis. A comparison of non-spatial (e.g. colour-shape) and spatial (e.g. colour-location) bindings would determine whether glucose operates selectively on tasks with a spatial component or more generally on binding processes. This is important because the hippocampal system supports a wide range of binding processes (Olsen et al. 2012) and while there is some evidence that glucose does not enhance the short-term maintenance of non-spatial (word-word) bindings (Stollery and Christian 2015), it would seem prudent to establish the proposed role of spatial memory in the observed enhancement. Thus, the possibility that spatial processing is an important feature of binding tasks that show short-term enhancement due to glucose remains to be determined. Additionally, examining binding efficiency changes over time would supply evidence relevant to the proposal that improved maintenance (e.g. slower decay rates or improved resistance to interference) underlies the improvement.

Several studies have reported evidence consistent with glucose producing a short-term increase in the efficient use of limited attentional resources (Benton et al. 1987, 1994; Flint and Turek 2003; Fucetola et al. 1999; Rao et al. 2005; Riby et al. 2008; Scholey et al. 2009; Serra-Grabulosa et al. 2010) and, given participants were explicitly required to remember object-location pairings, this could be one possible mechanism for improved binding. Specifically, our task requires the maintenance of multiple object-location pairs and participants may have strategically attended to a subset of these pairs by prioritizing resources. The impact of glucose presumably relates to locations because there are no reliable differences in the retention of the objects themselves. Under this scenario, there are two possible, but not mutually exclusive, processes whereby glucose could improve maintenance. The first is that glucose permits the active maintenance of a larger subset of locations. The second is that the subset of locations not actively maintained is less subject to degradation with glucose. Both processes permit improved binding efficiency, particularly if locations provide the binding nucleus. However, it remains unclear why glucose improvements should be equivalent for three object-location pairings, which should not tax attentional resources, and ten object-location pairings, which would. As such, therefore, this suggestion requires further scrutiny.

In connection with this, maintaining integrated information in the episodic buffer is also dependent on attentional resources (Baddeley et al. 2011), and reduced binding effectiveness of letter-location pairs is seen when a concurrent memory load is retained over a brief period (Elsley and Parmentier 2009). Even without a concurrent load, letter-location bindings decline rapidly during the first 5 s and then stabilize for at least another 10 s (Elsley and Parmentier 2015). These data suggest that attentional resources are critical for short-term maintenance. For comparison, the average reproduction times in our study ranged from 17 s (three items) to 37 s (ten items), making even our lowest memory load beyond this short-term maintenance period.

Other possibilities invoke known hippocampal functions. While the object-location task used here does not involve the complex spatial processes required for navigating environments, participants must utilize some form of egocentric or allocentric processing to retain the spatial layout of objects. While it is known that the hippocampus primarily supports allocentric processing (O'Keefe and Nadel 1978), both can cooperate to support performance (Burgess 2006; Ekstrom et al. 2014). In our study, egocentric processing would encode each object-location pair from the participant’s viewpoint. Allocentric processing would encode object-location pairs in relation to each other and establish a higher-order representation incorporating the configuration of locations. Finally, when the participant recalls the material, they must reorient from the vertical presentation format to the horizontal recall format. This description provides two possible mechanisms for how glucose might improve location memory and binding. First, transferring from the vertical presentation phase to the horizontal recall phase initiates attentional-updating process that can disrupt temporary object-location bindings (Golomb et al. 2014b). Thus, the superior binding following glucose may indicate a memory representation that is more resistant to disruption. Alternatively, preferential allocentric-based processing of the spatial array may create a memory representation that better preserves the configural properties of the display, with this viewpoint-independent representation better supporting location memory and, consequently, binding. Of course, since our participants were explicitly required to remember what objects went where and we tested memory using free-recall, rather than recognition, the conscious controlled recollection that this entails could provide another hippocampal-based process capable of sustaining binding. In this context, it is important to note that while location encoding may be automatic, the later retrieval of that location is clearly not. Given that recollective experiences are associated with more contextual knowledge and location appears to be a strongly encoded contextual feature when memorizing objects (Perfect et al. 1996), the importance of the hippocampal system to the encoding and retrieval of contextual features (e.g. location) could form a general basis for the findings and is consistent with other studies showing better recollection with glucose (e.g. Smith et al. 2009; Sunram-Lea et al. 2008).

As noted earlier, the hippocampal system lies at the centre of the core function of binding disparate elements together, and it is known that object-location memory shows complex cooperative interactions among several brain regions such as the hippocampus, perirhinal cortex, and the medial prefrontal cortex (see Barker et al. 2007; Barker and Warburton 2009, 2015; Warburton and Brown 2010). For example, there is good anatomical evidence for projections between the hippocampus and medial prefrontal cortex, medial prefrontal cortex and perirhinal cortex, and perirhinal cortex, via the entorhinal cortex, to the hippocampus (e.g. Brown and Aggleton 2001). Furthermore, lesions of the parahippocampal and fornix regions, two important hippocampal communication pathways, impair object-location memory (Bussey et al. 2000). More importantly for a neurochemical understanding of how glucose might improve performance, there seems to be a critical role for the cholinergic system within this circuit for object-location memory (Barker and Warburton 2009). Other work on object-location memory in rats has also shown that NMDA receptor antagonists and mACh antagonists impair memory, NMDA agonist and cholinesterase inhibitors improve memory, and inactivation of the CA1 hippocampal area blocks learning (Assini et al. 2009). These observations are consistent with one of the several possible mechanisms whereby glucose modulates episodic memory, that of facilitating acetylcholine synthesis (Messier 2004; Riby and Riby 2006).

In conclusion, the current study found that glucose improves the ability to remember the locations of objects and to bind objects to those locations. Based on current knowledge, this suggests a strong hippocampal basis and the findings are therefore consistent with the view that glucose has an affinity for hippocampal-based cognitive processing. How best to understand these improvements, and the extent spatial processing plays a critical role, requires further exploration as several suggestions are compatible with the current data. The asymmetric binding strength of the two bonds linking object and location, together with the notion that glucose might selectively enhance the updating of contextual detail, is clearly in need of further investigation. Nevertheless, our study highlights the usefulness of the object-location paradigm for progressing our understanding of the functional basis for glucose improvements in cognition. This is especially pertinent given that the improvements we found differentiate, using a single paradigm, three fundamental aspects of episodic memory: the “what”, the “where”, and the “what-was-where”.
